# Supplementary material for: A transition to stable one-dimensional swimming enhances E. coli motility through narrow channels
Source: Nat Commun. 2020 May 11;11:2340. doi: 10.1038/s41467-020-15711-0 (PMC7214458; doi:10.1038/s41467-020-15711-0)
Supplement: Supplementary file 1 — Supplementry Information [file 41467_2020_15711_MOESM1_ESM.pdf]

Supplementary Information for

A transition to stable 1D swimming enhances *E. coli* motility through narrow channels

Gaszton Vizsnyiczai, Giacomo Frangipane, Silvio Bianchi,
Filippo Saglimbeni, Dario Dell'Arciprete, and Roberto Di Leonardo

1 Supplementary Note 1: Size measurement of the microtunnels

We used two-photon fluorescence laser scanning microscopy to precisely measure the inner size of the SU-8 microtunnels. Since SU-8 has low intrinsic fluorescence, we have decided to cover and fill the microtunnel structure with unpolymerized SU-8 containing a high efficiency two-photon flourescence dye (Fig. 1). We have found that the dye coumarin-1 can be mixed with SU-8 in high concentration and it is highly fluorescent for 780 nm two-photon excitation.

Covering the SU-8 microtunnels with dye mixed SU-8 produces a sample where the refractive index is uniform, and only the spatial distribution of the fluorescent dye is unknown, ideal for precise size measurement with two-photon fluorescence scanning microscopy.

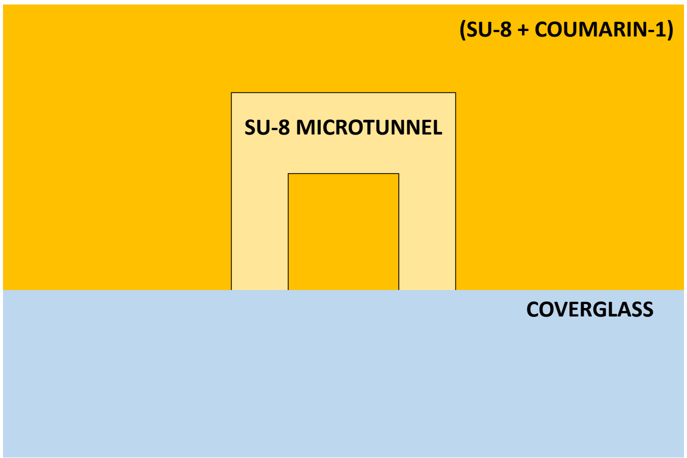

Supplementary Figure 1 Schematic side-view drawing of a microtunnel structure filled and covered with unpolymerized SU-8 mixed with coumarin-1 dye.

Coumarin-1 was mixed into SU-8 2015 in a concentration of 0.4 g/ml. The mixture was then baked at 95 °C to remove the thinner solvent of SU-8 by evaporation. This was necessary because we observed that polymerized microstructures deform, and the coumarin dye diffuse into them when they are covered with thinner diluted SU-8. The microtunnel structure was covered and filled with the dried out coumarin-SU-8 at 65 °C, which is above the glass transition temperature of the dry SU-8.

We have performed the two-photon fluorescence laser scanning microscopy on the optical setup of the two-photon polymerization system that fabricated the microtunnel structure. The only change in the system was the replacement of the imaging camera, that normally monitors the fabrication process, with a silicon avalanche photodetector (Thorlabs APD410A2/M).

Measurements were done by scanning a laser focus with 0.5 mW optical power in a plane perpendicular to the axis of the microtunnels. The two-photon excited fluorescence signal detected by the avalanche photodiode was registered in synchrony with the scanning movement of the focus. The resulting cross section images of the microtunnels are shown on Fig. 2.

From the recorded cross section images the lateral and vertical profile of each microtunnel was extracted. Widths and heights of the microtunnels then were determined by fitting their profile curves with a boxcar-function convolved by a numerically calculated two-photon point spread function, the result are reported in Table 1.

tunnel #	1	2	3	4	5	6	7	8
width	1.435	1.635	1.9	2.12	2.365	2.87	3.395	3.915
height	1.4	1.93	2.18	2.41	2.59	3.03	3.49	3.96
mean	1.4175	1.7825	2.04	2.265	2.4775	2.95	3.4425	3.9375

Supplementary Table 1 Measured width and heights of the microtunnels in micrometers.

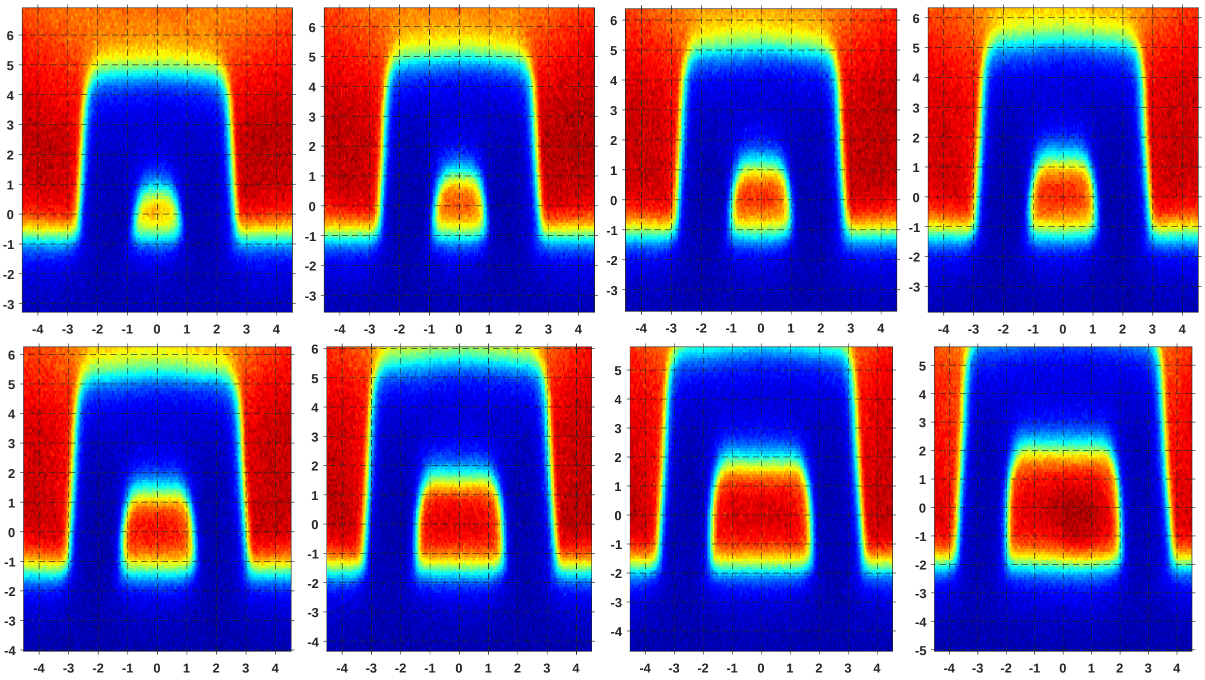

Supplementary Figure 2 Two-photon fluorescence cross section images of the microtunnels. Axes units are in micrometers.

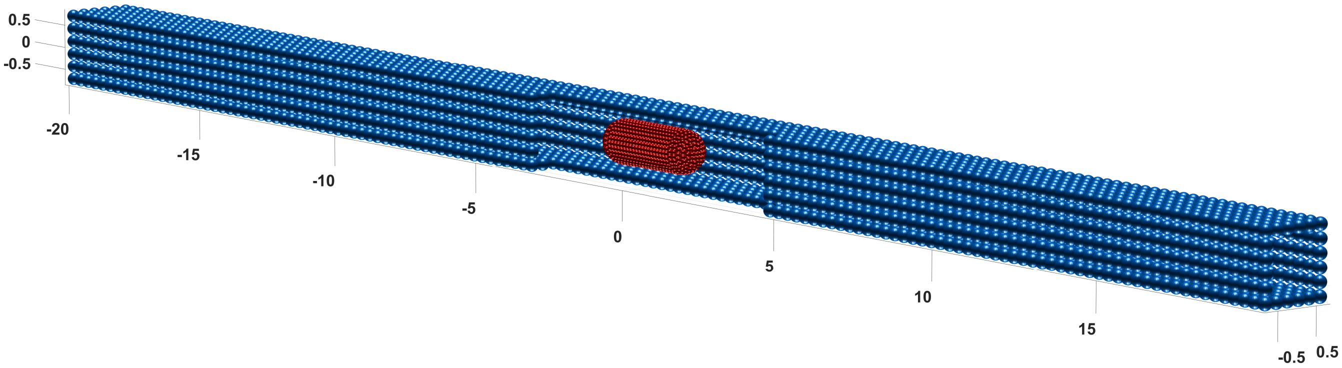

Supplementary Figure 3 Three-dimensional plot of the hydrodynamic model geometry of an *E. coli* cell body in a square microtunnel (1.42 μm width). A section of the microtunnel is not plotted to make the cell body inside visible. Axis units are in micrometers.

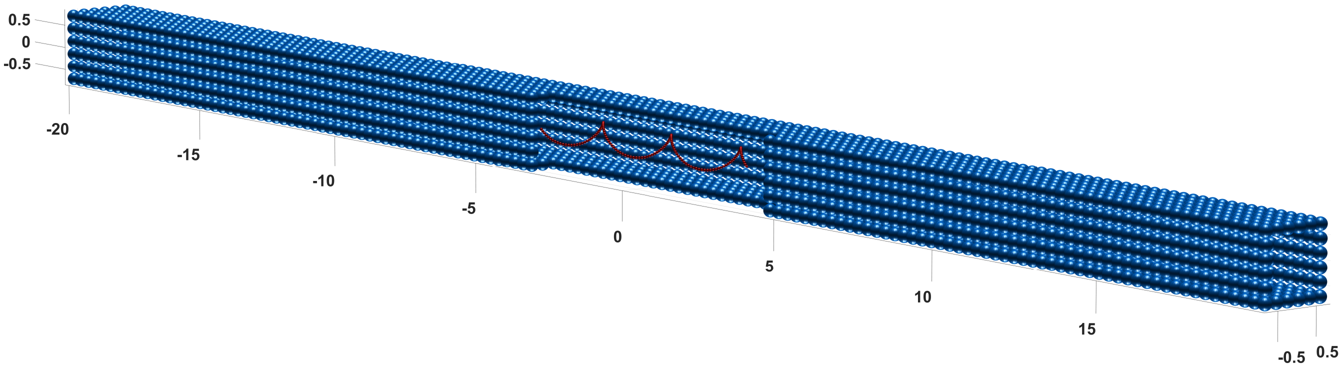

Supplementary Figure 4 Three-dimensional plot of the hydrodynamic model geometry of a helical flagella in a square microtunnel (1.42 μm width). A section of the microtunnel is not plotted to make the flagella inside visible. Axis units are in micrometers.

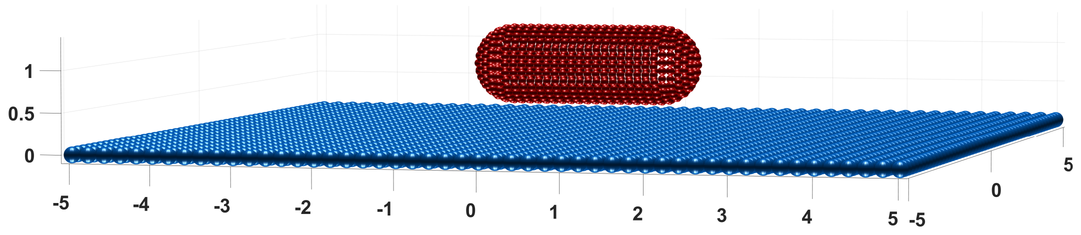

Supplementary Figure 5 Three-dimensional plot of the hydrodynamic model geometry of an *E. coli* cell body over a flat surface. Axis units are in micrometers.

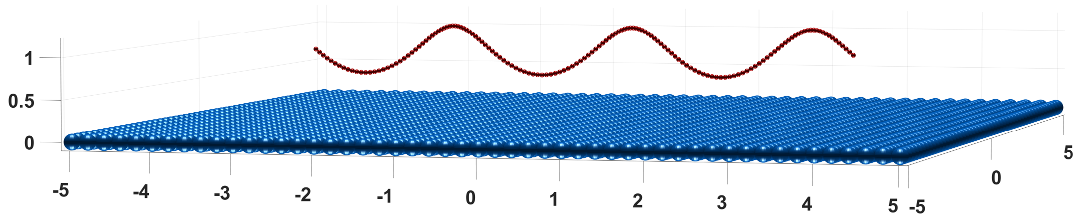

Supplementary Figure 6 Three-dimensional plot of the hydrodynamic model geometry of a helical flagella over a flat surface. Axis units are in micrometers.

2 Supplementary Note 2: simulations using the Rotne-Prager method.

In Rotne-Prager method rigid bodies are represented as clusters of close packed small spherical beads. We indicate with $\mathbf{v}_{\text{tot}}$ the $6 \times N$ vector containing the transnational and rotational velocities of the N beads composing our system. Similarly, we call $\mathbf{f}_{\text{tot}}$ the $6 \times N$ vector storing the forces and torques components acting on the beads. For a given configuration of beads a grand mobility matrix $\mathbf{M}$ is calculated:

$$\mathbf{v}_{\text{tot}} = \mathbf{M}\mathbf{f}_{\text{tot}} \tag{1}$$

To compute the drag coefficients of a rigid cluster of spheres we compute a $6N \times 6$ matrix, which we indicate with $\mathbf{C}$, connecting $\mathbf{v}_{\text{tot}}$ with the vector $\mathbf{v}_c = (v_{cx}, v_{cy}, v_{cz}, \omega_{cx}, \omega_{cy}, \omega_{cz})$ storing the linear and rotational velocity components of the cluster. More explicitly we have that:

$$\mathbf{v}_{\text{tot}} = \mathbf{C}\mathbf{v}_c \tag{2}$$

In our case, we want to compute the drag of a single rigid cluster (e.g. a spherocylindrical body) in the presence of fixed boundaries (the channel). All the entries of $\mathbf{C}$ connecting $\mathbf{v}_c$ with the spheres composing the channel are 0 to impose the condition of fixed boundaries. Incidentally the transpose of $\mathbf{C}$ is the matrix combining all the elements of $\mathbf{f}_{\text{tot}}$ to obtain the vector $\mathbf{f_c} = (f_{cx}, f_{cy}, f_{cz}, T_{cx}, T_{cy}, T_{cz})$ storing the force and torque components of the entire cluster:

$$\mathbf{f}_c = \mathbf{C}^T \mathbf{f}_{\text{tot}} \tag{3}$$

If we combine Eq. 1 with the Eq. 2 and Eq. 3 we obtain the following linear relation between $\mathbf{v}_c$ and $\mathbf{f}_c$:

$$\mathbf{f}_c = \mathbf{C}^T \mathbf{M}^{-1} \mathbf{C} \mathbf{v}_c \tag{4}$$

meaning that the drag matrix associated to the rigid cluster is $\mathbf{D}_c = \mathbf{C}^T \mathbf{M}^{-1} \mathbf{C}$. Fig. 3 and Fig. 4 show a representative configuration of the spheres in our Rotne-Prager simulations respectively for a cell body and for an helix in channel. The obtained drag coefficients of the two can be compared with their corresponding values when the cell is out of the channel and swims close to the coverglass interface as shown (see Fig. 5 and Fig. 6).

To simulate a swimming cell and check the stability of the channel axis we divide the bacterium into two parts: the cell body having 6 degrees of freedom (3 translations and 3 rotations) and the flagellar bundle that is rigidly anchored to the cell body but can freely rotate around its axis. Calling ω_f the rotational speed of the flagellar bundle around its axis, the velocity has now has 7 components i.e. $\mathbf{v}_c = (v_{cx}, v_{cy}, v_{cz}, \omega_{cx}, \omega_{cy}, \omega_{cz}, \omega_f)$. According to this picture we also add to $\mathbf{f}_c$ an extra component T_f for the torque applied to the flagellar bundle. After computing $\mathbf{D}_c$ we can invert it and obtain the cluster mobility $\mathbf{M}_c = \mathbf{D}_c^{-1}$. The flagellar motors apply opposite torque on the bundle and on the body so that, if the cell axis is aligned along the z direction, we have that $\mathbf{f}_c = (0,0,0,0,0,T,-T)$. The result velocity can be simply obtained as $\mathbf{v}_c = \mathbf{M}_c \mathbf{f}_c$.

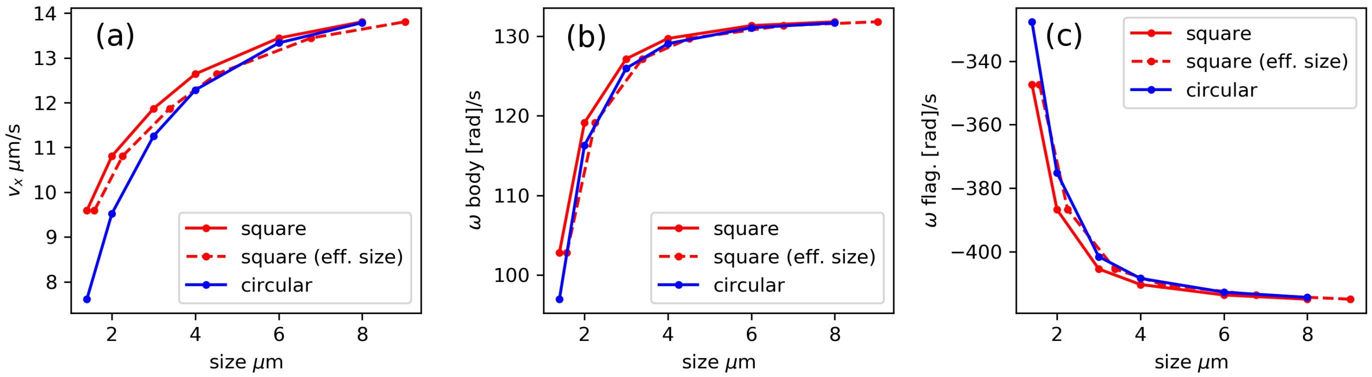

Supplementary Figure 7 A cell placed at the center of channels with circular and square sections. The velocity component along the channel axis (a), rotational speed of the body (b) and the rotational speed of the flagellar bundle (c) are plotted as a function of the channel size. For circular channels (blue) the size is given by the diameter while for a square channel (red) the size is the side of the square. Red dashed line plots the same speeds of the square channel as a function of its effective diameter that is the square side multiplied by a factor $\sqrt{4/\pi}$ (see text).

We simulate the cell in square channels with the same size of the experimental ones. Both the cell and the channel axes are oriented along the z direction. In Fig 7 we plot the cell speed as a function of the channel size when the cell is at the center of the channel. As expected, we observe no speed increase since the cells are placed at the center of the channel. We also simulate cells in circular channels with diameters equal to the square channel size. Compared to the circular channel, the square channel displays approximately the same curve if we plot the linear and rotational speeds as a function of an effective diameter that is the diameter of an equivalent circular channel with same section area (i.e. the square size multiplied by $\sqrt{4/\pi}$). To check the swimming stability we displace the cell from the channel axis by Δx and look at its corresponding velocity component v_{cx}. In Fig. 8 we plot v_{cx} as a function of Δx. If v_{cx} is negative for a positive Δx the cell is pulled back towards the center and the cell remains in proximity of the cell axis. A transition from stable to unstable swimming is observed at a channel size of 2.5 μm. A similar behaviour is observed in circular channels. In the text we show that a the values of dv_{cx}/dx at $\Delta x = 0$ are quantitatively similar if the square and the circular channel have the same section area.

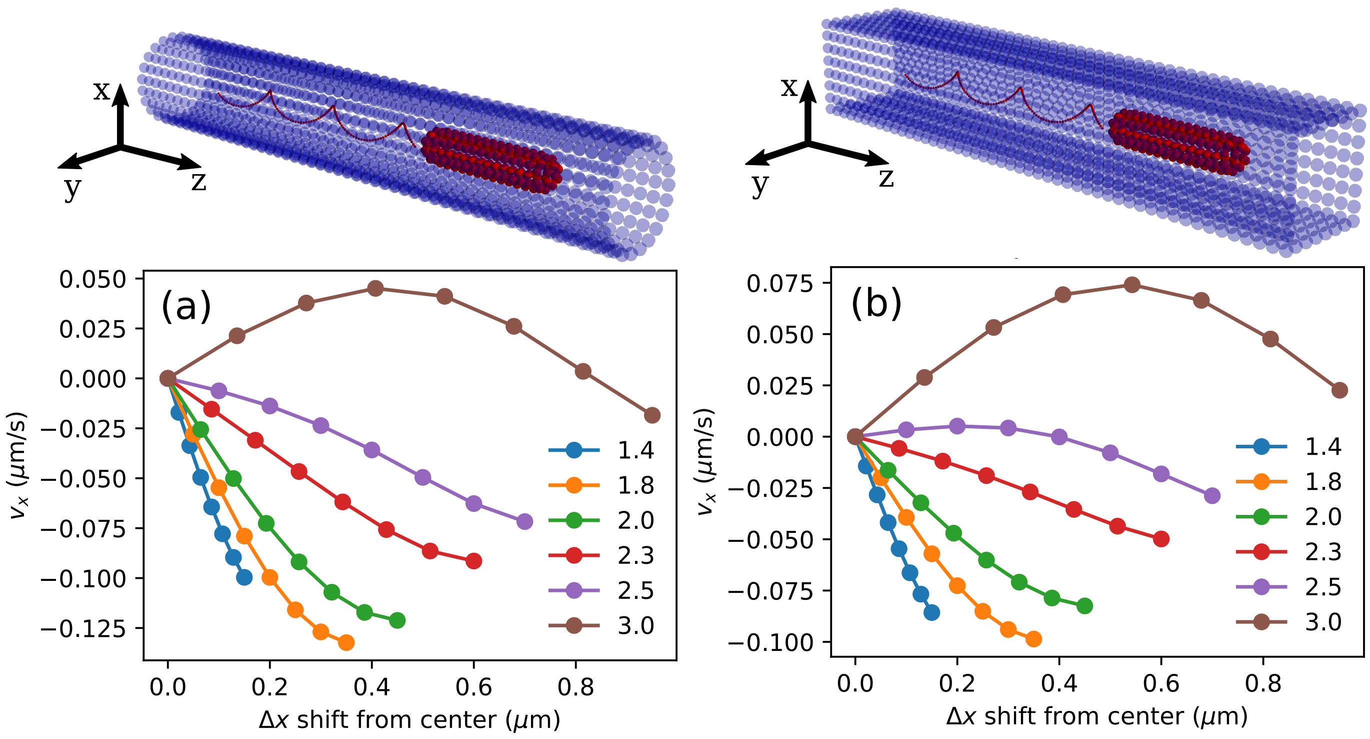

Supplementary Figure 8 Velocity component (v_x) of the cell pointing toward the channel center as a function of its displacement. (a) Circular channels with diameters ranging from 1.4 to 3 μm . (b) Square channels with sizes ranging from 1.4 to 3 μm.

3 Supplementary Note 3: Cell body rotation of cells going out of the last channel.

Fig. 5 in the main text shows that the cell velocity peaks when the cell body is out of the last tunnel and the flagellar bundle is still inside the tunnel and then relaxes to the "free" speed value after the flagella are completely out of the tunnel. In the theoretical model established to explain the observed speed curves we assume a constant flagellar torque based on literature reported data. Here we provide further experimental evidence in support of the constant torque assumption. Fig. 9 plots the body angle as function of time of several cells coming out from the tightest channel. Time origin is shifted so that for $t < 0$ (shaded area) the cell body is inside or partially inside the tunnel. Conversely, for $t > 0$, the cell body is completely out of the tunnel while the bundle is progressively coming out. The transition from confined to free cell body is accompanied by an increase of the wobbling amplitude. Each orange line in Fig. 9 fits with constant frequency oscillations the cell angle dynamics soon after the cell body is completely out. When the cell body is completely out of the tunnel, the rotational drag of the body B_0 is constant therefore a constant frequency corresponds to a constant torque ($\Omega = T/B_0$). For $t > 0$ the bundle is coming out of the last tunnel so that its rotational drag is changing as also its rotational frequency. We can then conclude that the torque remains constant despite the fact that bundle frequency should vary confirming that the flagellar motors are working in a constant torque regime.

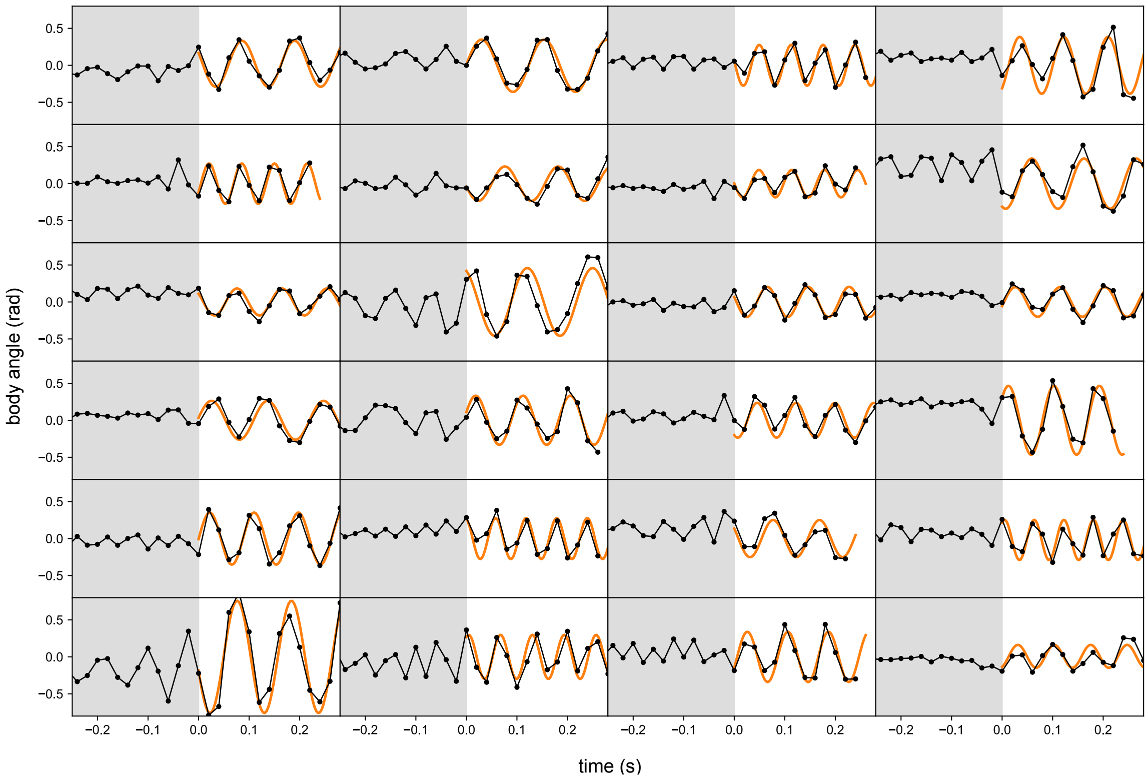

Supplementary Figure 9 Cell body orientation of several cells coming out of the last channel. The time origin is shifted so that at $t = 0$ the full cell body is out of the tunnel. Orange lines represent the best fit obtained for a sinusoidal function oscillating with constant frequency.

4 Supplementary Note 4: Measurements and correlations of *E. coli* swimming along the channels

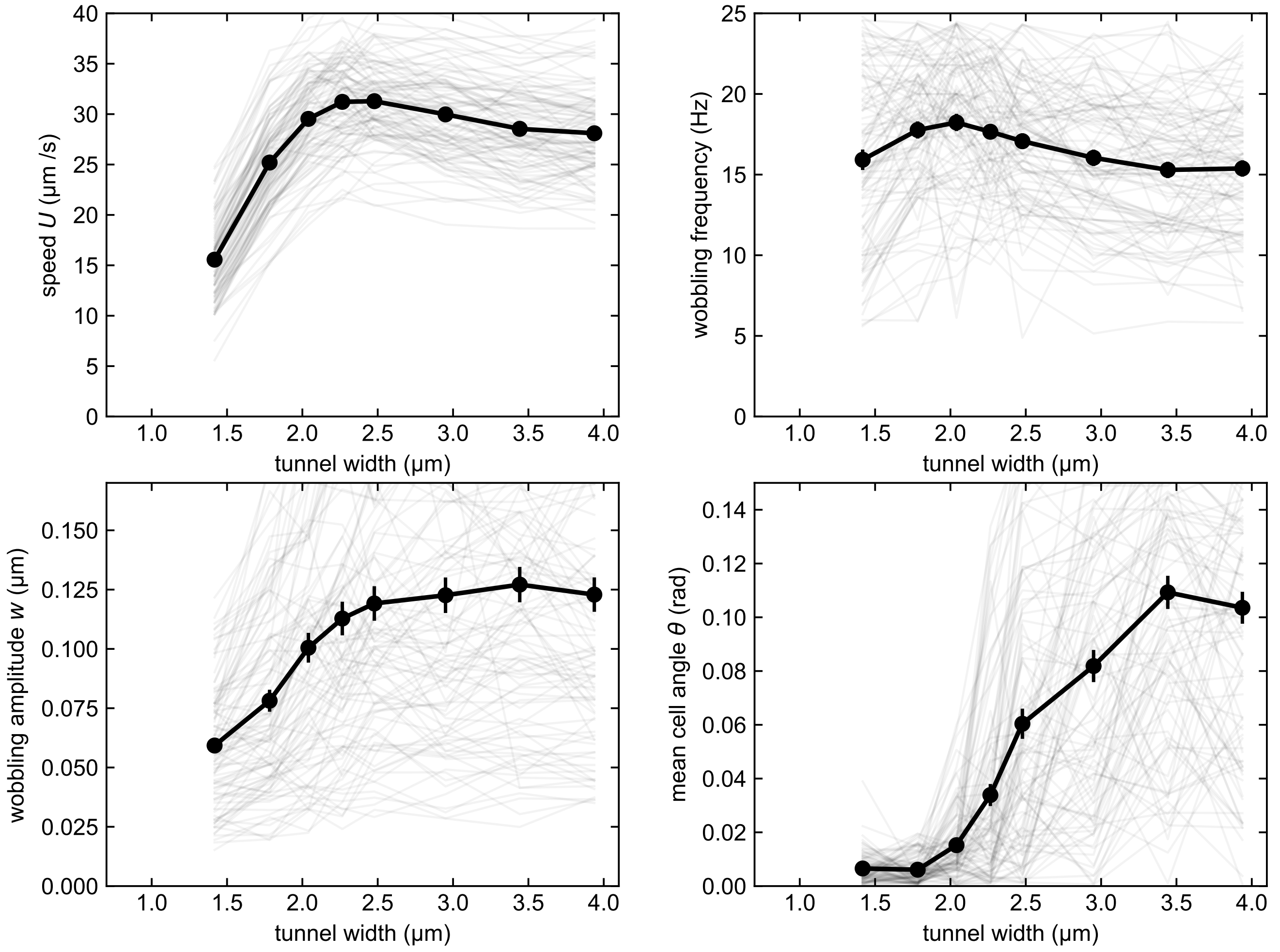

Supplementary Figure 10 Swimming speed, wobbling frequency, wobbling amplitude w, mean cell angle θ (as defined in Fig.3a) through tunnels of different diameter. Error bars represent SEM. Where Error bars are not visible are within the symbol size.

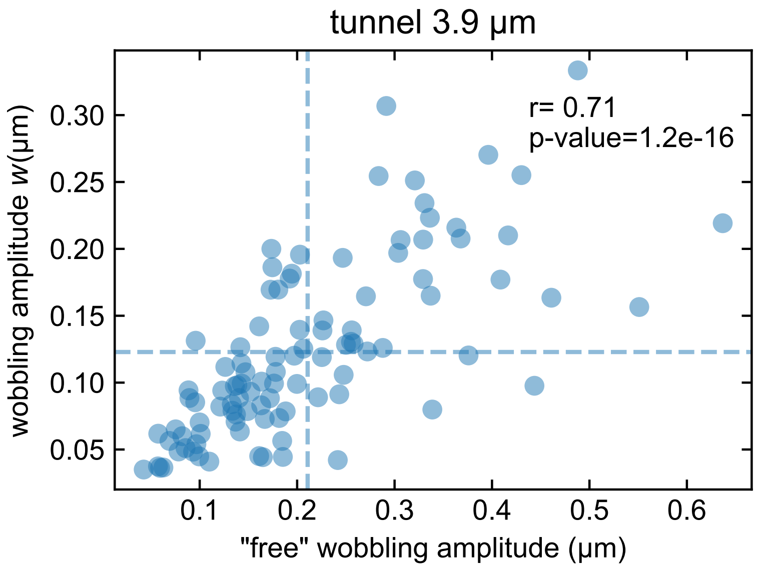

Supplementary Figure 11 Correlations between wobbling amplitude on the surface ("free") and in the largest tunnel. Dashed lines represent mean values.

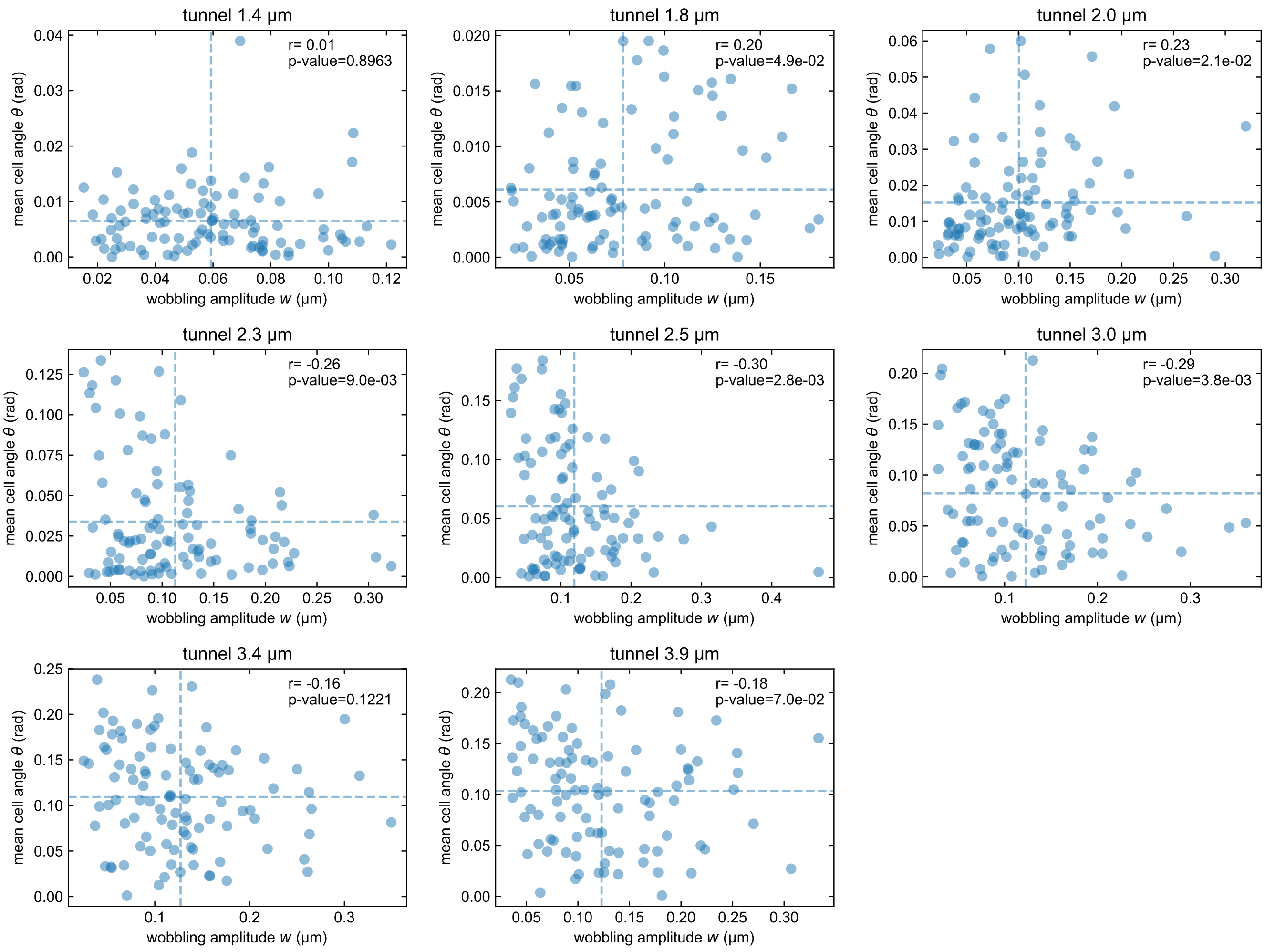

Supplementary Figure 12 Correlation between cell angle θ and the wobbling amplitude in all the tunnels. Dashed lines represent mean values.
