# Supplementary material for: A transition to stable one-dimensional swimming enhances E. coli motility through narrow channels
Source: Nat Commun. 2020 May 11;11:2340. doi: 10.1038/s41467-020-15711-0 (PMC7214458; doi:10.1038/s41467-020-15711-0)
Supplement: Supplementary file 3 — Description of Additional Supplementary Files [file 41467_2020_15711_MOESM3_ESM.pdf]

Description of Additional Supplementary Files

Supplementary Movie 1

An E. coli cell is funneled into a microstructure consisting of a sequence of tunnels of decreasing size. In the first four tunnels the cell is constantly in contact with a wall. Axial swimming becomes stable from the 5th tunnel where swimming speed is maximal.
